# Identification of a “Blue Zone” in the Netherlands: A Genetic, Personal, Sociocultural, and Environmental Profile

**DOI:** 10.1093/geront/gnae132

**Published:** 2024-09-12

**Authors:** Dorly J H Deeg, Theo van Tilburg, Marjolein Visser, Arjan Braam, Najada Stringa, Erik J Timmermans

**Affiliations:** Department of Epidemiology and Data Science, Amsterdam University Medical Centers, Vrije Universiteit, Amsterdam, North-Holland, The Netherlands; Amsterdam Public Health Research Institute, Amsterdam, North-Holland, The Netherlands; Department of Sociology, Vrije Universiteit, Amsterdam, North-Holland, The Netherlands; Amsterdam Public Health Research Institute, Amsterdam, North-Holland, The Netherlands; Department of Health Sciences, Faculty of Science, Vrije Universiteit, Amsterdam, North-Holland, The Netherlands; Department of Humanist Chaplaincy Studies for a Plural Society, University of Humanistic Studies, Utrecht, The Netherlands; Department of Emergency Psychiatry and Department of Residency Training, Altrecht Mental Health Care, Utrecht, The Netherlands; Department of Epidemiology and Data Science, Amsterdam University Medical Centers, Vrije Universiteit, Amsterdam, North-Holland, The Netherlands; Amsterdam Public Health Research Institute, Amsterdam, North-Holland, The Netherlands; Department of Epidemiology and Data Science, Amsterdam University Medical Centers, Vrije Universiteit, Amsterdam, North-Holland, The Netherlands; Julius Center for Health Sciences and Primary Care, University Medical Center Utrecht, Utrecht University, Utrecht, The Netherlands

**Keywords:** Environmental livability, Genetics, Lifestyle, Longevity, Social connectedness

## Abstract

**Background and Objectives:**

“Blue Zones” (BZs) are regions with exceptionally high numbers of longevous inhabitants. Several factors have been suggested to promote longevity in BZs, but the evidence generally does not meet scientific quality criteria. We aimed to characterize a municipality as a “relative BZ,” satisfying 3 criteria: compared to other municipalities, more exceptionally longevous inhabitants, a higher life expectancy, and a more stable population.

**Research Design and Methods:**

The population-based Longitudinal Aging Study Amsterdam has been ongoing since 1992 in 11 municipalities across the Netherlands with 3- or 4-yearly measurement waves. Using all available waves, we included 39 genetic, personal, sociocultural, and environmental characteristics.

**Results:**

One municipality satisfied the 3 BZ criteria. In comparison with participants in other municipalities in the same province and other provinces in the Netherlands, BZ-participants more often had a polygenic risk score linked to longevity, smoked less, consumed less alcohol and more fruit, biked more minutes, did more often paid work, practiced singing more often, attached higher importance to religion, and lived in a more walkable and livable environment. In contrast, BZ-participants had a slower walking speed, more depressive symptoms, felt less purpose in life, had a larger waist circumference, walked and did sports less often, consumed less vegetables, and exchanged less instrumental support. Other indicators of their physical and mental health and social connectedness did not substantially differ from non-BZ-participants.

**Discussion and Implications:**

Rather than clues to healthy aging, our findings suggest factors conducive to longevity regardless of impaired health.

## Background and Objectives

“Blue Zones” are regions in the world, mostly remote, where inhabitants share exceptional longevity as commonly evidenced by a high number of centenarians (Poulain et al., 2013). The “Blue Zone” (BZ) concept is popular because BZs are considered to provide clues to healthy aging (Buettner & Skemp, 2016). The supposedly high number of very old inhabitants of BZs has been attributed to a series of health and lifestyle factors, of which healthy diets and high physical activity are most prominent. A range of additional behavioral factors has been forwarded, including low smoking rates, low prevalence of obesity, an agricultural occupation, a relaxed daily pattern including mid-day napping, a low prevalence of depression, strong religious faith, social connectedness, and respect towards elders (Fastame et al., 2021; Panagiotakos et al., 2011; Poulain et al., 2021). Also, biological factors have been reported, including specific genes such as PON1-rs662 and TAS2R38 (Melis et al., 2019; Poulain et al., 2021) and a superior immune system (Soloski et al., 2022), as well as factors that constitute a healthy environment, for example, a mountainous area inviting exercise (Herbert et al., 2022).

Interestingly, other environmental factors have been found that are not directly associated with healthy behavior, social connectedness, or genetics. Tolu et al. (2019) reported that in the Ogliastra region in Sardinia, Italy, an established BZ, a high prevalence of hypothyroidism is observed, due to a low exposure to natural iodine. Hypothyroidism has been shown to be associated with greater longevity (Rozing et al., 2010). Chrysohoou et al. (2013) reported a relatively high level of natural radioactive radiation in another established BZ, Ikaria, Greece. Low-dose radiation has been shown to be associated with reduced inflammation (Rödel et al., 2012). The evidence on these environmental factors sheds doubt on the causal relation between healthy behaviors and longevity in BZs.

Regarding all these factors, there is relatively little research that meets the usual scientific quality criteria. First, the proportion of centenarians depends on the denominator used. Several studies count the number of centenarians at a certain point in time and divide this count by the number of newborns around the time of birth of the current centenarians (Poulain et al., 2013). However, these two populations do not necessarily belong to the same cohort, because out- and in-migration may have occurred. Furthermore, many studies have small sample sizes, because BZs are small and sparsely populated (e.g., Fastame et al., 2021). Furthermore, the number of centenarians likely fluctuates over time (Huang & Jaquez, 2017). Because the absolute numbers of centenarians are small and fluctuating, most studies resort to samples of nonagenarians or younger adults (e.g., Pes et al., 2020). Moreover, many studies lack a comparison group (e.g., Legrand et al., 2019). Some studies adopt an ecological approach, the conclusions of which are not directly translatable to individual behavior (e.g., Pes et al., 2013).

Since the launch of the “Blue Zones” concept, it has been criticized. An early criticism is that in the regions identified as BZs, illiteracy is high and the self-reporting of age is far from exact (Gavrilov & Gavrilova, 2019; Newman, 2018). Furthermore, population registers are far from perfect. Another criticism has been voiced recently, arguing that the count of inhabitants aged 100 and over may or may not be correct, but the life expectancy in this region should be concomitantly high. However, it has been demonstrated that the life expectancy in BZs is relatively low (Newman, 2019). Conceptual criticism is possible as well. From the observation of BZs, characteristics are derived that are considered to promote longevity, thereby implying that these characteristics are universal (Buettner & Kemp, 2016). Several studies, however, show evidence of heterogeneity in factors contributing to longevity across individuals, time, and contexts (Fernández-Ballesteros et al., 2022; Noale et al., 2005). Furthermore, as a societal phenomenon, the recent increase in popularity of BZs has been criticized as reflecting a “neoliberalization of public health,” because the concept promotes an apolitical discourse on individual responsibility and ownership of health (Carter, 2015). The emphasis on “nudging” individuals towards healthy behaviors through small changes in the local environment is considered a reflection of “libertarian paternalism,” a variant of neoliberalism (Carter, 2015).

In view of the attractiveness of the “Blue Zones” concept on the one hand, and the various criticisms of the concept, on the other hand, research on this concept using high-quality data is needed. A first prerequisite is the existence of a population registry that allows reliable age and vital status ascertainment. This is available in the Netherlands. A second prerequisite is the availability of data on a wide range of health and social characteristics that have been linked to BZs. The Dutch interdisciplinary, population-based Longitudinal Aging Study Amsterdam (LASA) has been ongoing since 1992 in 11 municipalities across the Netherlands from which random samples were recruited (Hoogendijk et al., 2020). Linkage with the population registry provides the vital status of the participants. It allows the ascertainment of the cumulative number of exceptionally longevous participants across a study period of over 27 years, which is deemed to be more stable than the number observed in a specific year. The availability of participants’ vital status also allows the ascertainment of the average life expectancy in each municipality, which we include as an additional criterion to identify a Blue Zone in line with Newman’s (2019) criticism. A third prerequisite is the possibility to determine the stability of the population in each municipality. If the population is relatively unstable, the number of exceptionally longevous participants may be confounded by in-migration of long-lived people or out-migration of short-lived people (Poulain et al., 2013), so that longevity cannot be considered to be a characteristic of the municipality. Based on the addresses of LASA participants, the stability of the population in each municipality can be determined by tracking the migration into and out of it. These three prerequisites are examined in the first part of our study, addressing the research question:

1. Can a municipality be identified that satisfies all three criteria to define a relative BZ: among the 11 municipalities, the highest cumulative number of exceptionally longevous participants, the highest life expectancy, and the most stable population?

In the second part of our study, presuming that a BZ can be identified, the BZ is characterized in comparison with other municipalities, addressing the research questions:

2a. How do the inhabitants of a BZ compare to inhabitants of municipalities elsewhere regarding genetics, physical and mental health, lifestyle, social connectedness, and religiousness?
2b. How do the environmental characteristics of a BZ compare to those of municipalities elsewhere?

## Research Design and Methods

### Data

Longitudinal Aging Study Amsterdam is an ongoing longitudinal study with baseline measurement waves in 1992/1993 and 3- or 4-year follow-up waves (Hoogendijk et al., 2020). Its sample was recruited from the municipal registries in 11 municipalities in three geographic regions that together represent the sociocultural variety in the Netherlands: the Protestant North-East (5 municipalities), the Roman Catholic South (3 municipalities), and the secularized West (3 municipalities). Each region included a larger city and smaller municipalities, together representing the distribution of urban and rural areas across the Netherlands. The sample’s initial ages were 55–85 years, with oversampling of higher ages and men, resulting in 48.5% men. The baseline cooperation rate was 62%, resulting in 3,107 participants. Data collection involved interviews in the participants’ homes and self-completion questionnaires. Informed consent was obtained from every participant. In 2002/2003 and 2012/2013, new cohorts aged 55–64 years were recruited from the same sampling frame as in 1992/1993, with 1,002 and 1,023 participants, respectively.

As only participants from the first cohort can have reached exceptional longevity during the study period, the criteria to identify a BZ are examined in this cohort. For the comparison of characteristics of BZ and other regions, data from all three cohorts and all available waves are used (5,132 participants). Average wave-to-wave all-cause attrition from the study sample amounted to 18.0%; average wave-to-wave non-mortality attrition was low at 5.8% and differed statistically significantly neither between municipalities nor between waves (Supplementary Table 1). Year of birth and years of education were associated with non-mortality attrition and thus were included in all analyses for part two of our study.

### Measures: Criteria for a Blue Zone

#### Exceptional longevity count

The first criterion for a BZ, a high share of exceptionally longevous participants, was ascertained by following the first cohorts’ vital status in the official population registry from baseline through December 2019. The ascertainment of vital status was 99.7% complete. Exceptional longevity was defined as having reached age 95 or over because in our cohort, the number of participants who reached the age of 100 was still very small (*n* = 26). Each participant’s age in December 2019, if alive, or age at death was determined. For each municipality, the share of exceptional longevity was then calculated as the proportion of participants aged 95 years or over (numerator) among all participants, alive or deceased (denominator).

#### Survival time

The second criterion, a high life expectancy, involved the determination of the survival time of first-cohort participants (ages 55+), using the Realized Probability of Dying (RPD, Deeg et al., 1989). The RPD is a relative measure of longevity that compares each individual’s survival time, according to year of birth and sex, with the total Dutch population’s survival time from the LASA baseline onwards. This is done using cohort life tables based on the total Dutch population for successive years during the study period. RPD values range from 0 to 1. RPD values below 0.50 indicate longer survival compared to the median population; values above 0.50 indicate shorter survival. For example, the value of a man’s RPD is 0.80 if, at the time of his death, 80% of his baseline cohort is still alive. At the end of mortality follow-up (December 31, 2019), 15% of the first study cohort was still alive. The RPD for these participants is estimated by assuming that their remaining survival time corresponds to the median population survival time from the end of follow-up onward. This amounts to multiplying the probability of reaching the end of 2019 by 0.5.

As the RPD has a uniform distribution, it is transformed to a standard normal distribution using the logit of the RPD (LRPD), where lower values indicate greater relative survival. The LRPD is averaged across participants in each municipality.

#### Stability of the population

The third criterion, the stability of each municipality’s population, involved, first, the proportion of participants who were born in this municipality and second, the proportion of moves out of this municipality during the full study period (1992–2019). First, at baseline (1992/1993) the participants were asked in which municipality they were born. When this was different from the municipality of residence at baseline, a participant was considered to have relocated from outside the municipality. Second, at each follow-up wave, it was established whether a participant had moved outside the baseline municipality or not.

### Measures: Characteristics

The measures selected for this study encompass a wide range of characteristics that are known to predict longevity, and include genetics, health, lifestyle, social connectedness, and environmental livability (Goldman et al., 2017; Poulain et al., 2021). For a detailed description, the reader is referred to the Supplementary Material. Selected characteristics that closely reflect the nine BZ ‘principles’ described by Buettner and Skemp (2016) are summarized in Table 1: (a) Move naturally, (b) Sense of purpose, (c) Downshifting stress, (d) 80% Rule: stop eating when the stomach is 80% full, (e) Plant slant: eat vegetables and legumes, (f) Wine: drink moderately, (g) Belong to a faith-based community, (h) Loved ones first: commitment to family, and (i) Right tribe: social circle supports healthy behavior.

**Table 1.** Selection of Characteristics That Are Approximate Operational Definitions of Blue Zone “Principles” (Buettner & Skemp, 2016)

“Principle”	Approximate operational definition
1) Move naturally	*Life style:* time spent walking and bicycling; *Environment:* walkability
2) Sense of purpose	*Health:* Valuation of Life subscale “Ambition”
3) Downshifting stress	*Health:* depressive symptoms
4) 80% rule	*Life style:* body mass index, waist circumference
5) Plant slant	*Life style:* frequency of consuming fruits and vegetables
6) Drink moderately	*Life style:* alcohol consumption
7) Faith-based community	*Social connectedness and participation:* church attendance, strong faith deemed important, praying
8) Loved ones first	*Social connectedness:* given and received social support
9) Right tribe	*Life style:* social circle stimulating a healthy lifestyle

### Statistical Analysis

To address the first research question, the 11 municipalities in our sample were examined regarding the three criteria for qualifying as a BZ. The municipality that ranked highest on all three criteria was defined as a BZ, relative to the municipalities sampled.

For the second research question, a series of regression models compared the characteristics of BZ with two other regions, that is, other municipalities in the same province (Rest of Province, RP) and in other provinces in the Netherlands (OP). The two comparisons were performed to achieve greater robustness. In addition, if a BZ characteristic was less favorable than RP but more favorable than OP, the characteristic was deemed neither favorable nor unfavorable. In each regression model, one characteristic was the dependent variable. The main independent variable was a categorical variable denoting BZ and the two other regions. Its reference category was BZ so that for each characteristic, two comparisons were made: (a) BZ to RP and (b) BZ to OP. In each model, basic covariates were sex, year of birth, and years of education. Analyses of physical activity were additionally adjusted for season and whether the past two weeks were normal or not. In order to reduce measurement error and to incorporate development in area characteristics over time, each characteristic was accumulated across as many LASA waves as were available for this characteristic. Twenty characteristics were measured at eight waves, while 15 characteristics were measured at fewer waves, ranging from seven to one (Genetics and Social circle stimulating healthy lifestyle). If for a characteristic only one wave was available, ordinary linear or logistic regression models were run; if for a characteristic more waves were available, Generalized Estimating Equations (GEE) with exchangeable correlation matrix were used to account for the interdependency of measurements. GEE provides estimates that combine between-subject and within-subject associations (Twisk, 2003). The dependent and main independent variables were time-varying, whereas the basic covariates were time-invariant and the additional covariates for physical activity were time-varying. A characteristic of BZ was considered significantly more or less favorable if the marginal estimate for BZ fell outside the 95% confidence intervals of the marginal estimates of both RP and OP and the difference was in the same direction.

Effect sizes for continuous dependent variables were calculated as the regression coefficient of independent dummy variables comparing RP to BZ and OP to BZ, divided by the standard deviation of the dependent variable, and denoted as beta. Absolute values 0.2–0.5 indicated a small, 0.5–0.8 a medium, and above 0.80 a large effect (Cohen, 1988). For dichotomous dependent variables, effect sizes were expressed as odds ratios (ORs), with values 1.20–1.50 or 0.67–0.83 considered to indicate a small, 1.50–1.80 or 0.56–0.67 a medium, and above 1.80 or below 0.56 a large effect.

## Results

### Identification of a Blue Zone

Clearly, one municipality, N1, ranks best on all three criteria (Table 2, last four columns). Notably, none of the other municipalities comes very close to N1 on either criterion. N1 has a cumulative proportion of exceptional longevous participants of 8.3% and a relative survival time (LRPD) of −0.127. The latter corresponds to an RPD of 0.468. For a man aged 65 years, this value amounts to a 10-month longer life expectancy than the Dutch population median, that is, 17.4 instead of 16.5 years. For a woman aged 65 years, this value amounts to a 9-month longer life expectancy, that is, 22.3 instead of 21.5 years. The percentage of participants who were born in the same municipality was highest in N1 at 68%. The percentage of stayers in their baseline municipality during the study period was also highest in N1 at 97%. In conclusion, municipality N1 fulfills all three criteria for a BZ, relative to 10 other municipalities across the Netherlands. In the following, we indicate municipality N1 by BZ.

**Table 2.** Characteristics of the 11 LASA Municipalities: Size of Population, Sample and Sample Density, Cumulative Percentage Participants Who Reached Ages of 95 and Over, Relative Survival Time, and Stability of the Population

Municipality code	Population in 1992^b^	*N* in LASA baseline sample	Sample density (%)	Cumulative % 95+^c^	Relative survival time: LRPD^d^	Stability from birth to baseline^e^	Stability since baseline^f^
N1	8,003	96	1.2	8.3^a^	−0.13^a^	67.7^a^	96.8^a^
N2	7,103	94	1.3	5.3	−0.01	19.1	92.4
N3	18,147	252	1.3	7.1	−0.07	36.1	88.9
N4	4,398	161	3.7	5.6	−0.01	42.9	90.6
N5	97,131	353	0.4	7.1	+0.04	33.1	92.63
W1	713,407	856	0.1	7.5	+0.11	57.3	87.3
S1	8,787	146	1.7	4.1	+0.05	35.4	93.2
S2	52,132	371	0.7	6.2	+0.16	33.2	92.55
W2	17,743	269	1.5	5.9	−0.06	22.7	88.0
S3	36,329	214	0.6	6.5	+0.12	29.9	93.4
W3	14,011	276	2.0	5.4	+0.04	32.1	86.9

*Notes*: LASA = Longitudinal Aging Study Amsterdam; LRPD = Logit of the Realized Probability of Dying.

^a^Ranks highest.

^b^Source: Statistics Netherlands.

^c^Source: LASA linked to vital statistics, 1992–2019.

^d^Logit of the Realized Probability of Dying; source: LASA linked to vital statistics, 1992–2019.

^e^Correspondence between place of birth and municipality at baseline; source: LASA, 1992/93.

^f^Correspondence between municipality at baseline (1992/93) and end of follow-up (2018/19) for the cohort recruited in 1992.

### The Blue Zone Compared to Other Regions of the Netherlands

Regarding the covariates, BZ did not differ significantly from other municipalities in terms of sex composition, but on average BZ-inhabitants were born 1.8 years later than inhabitants of other provinces (OP) and had a 1.5 years shorter education than inhabitants both in other municipalities in the same province (Rest of Province, RP) and in OP (Table 3).

**Table 3.** Comparison^a^ of Sociodemographic Covariates Across Regions, for All Participants (Three Cohorts)

	Blue Zone (BZ) (*n* = 168)	Rest of province (RP) (*n* = 1,487)	Other provinces (OP) (*n* = 3,477)
Sex: % (95% CI) female	52 (44; 59)	54 (51; 56)	51 (49; 53)
Year of birth, *M* (95% CI)	1933.2 (1931.0; 1935.4)^◊^	1933.1 (1932.4; 1933.9)	1931.5 (1931.0; 1931.9)
Education in years, *M* (95% CI)	8.0 (7.5; 8.5)^○◊^	9.5 (9.4; 9.7)	9.8 (9.6; 9.9)

*Notes*: CI = confidence interval, *M* = mean.

^a^Indications of the significance of differences between the Blue Zone and other regions are based on comparison of the estimate of the Blue Zone with the confidence intervals of the other regions, as follows: ^○^ Blue Zone differs from Rest of province; ^◊^ Blue Zone differs from Other provinces.

In Table 4, each row represents one GEE model. In the first category, Genetic make-up, the unfavorable ApolipoproteinE-epsilon4 was significantly more prevalent in BZ than in OP, but the favorable ApolipoproteinE-epsilon2 as well as the polygenic risk scores with and without ApolipoproteinE variants were significantly more often present in BZ than elsewhere. Effect sizes were (very) small, with beta = 0.10–0.14 for the polygenic risk scores and odds ratio (OR) = 1.25–1.52 for ApolipoproteinE-epsilon2 and -epsilon4.

**Table 4.** Characteristics of Blue Zone Compared to the Rest of its Province and Other Provinces, for All Observations Across All Waves Available: Marginal Means (Confidence Interval)^a^^,^^b^ and Effect Sizes

	Blue Zone (BZ) (*n* ≤682)	Rest of province (RP) (*n* ≤6,282)	Other provinces (OP) (*n* ≤13,995)	Effect size^c^ BZ—RP	Effect size^c^ BZ—OP
Genetic make-up longevity					
ApoE e2,^d^ % (95% CI)	22 (13; 32)^**○◊**^	16 (13; 19)	18 (16; 21)	**OR = 1.45**	**OR = 1.25**
ApoE e4,^d^ % (95% CI)	33 (21; 44)^**◊**^	33 (29; 36)	28 (25; 31)	OR = 1.28	**OR = 1.52**
Polygenic risk score without ApoE,^d^*M* (95% CI)	0.19 (−0.05; 0.44)^**○◊**^	0.02 (−0.06; 0.11)	−0.03 (−0.09; 0.03)	**beta = −0.10**	**beta = −0.14**
Polygenic risk score with ApoE,^d^*M* (95% CI)	0.20 (−0.05; 0.44)^**○◊**^	0.01 (−0.07; 0.09)	−0.02 (−0.08; 0.04)	**beta = −0.10**	**beta = −0.12**
Physical and mental health					
Self-rated health,^e^ range 1–5 = worst, *M* (95% CI)	2.42 (2.32; 2.53)	2.35 (2.32; 2.39)	2.47 (2.44; 2.49)	beta = 0.07	beta = −0.04
Gait speed in m/s,^e^*M* (95% CI)	0.81 (0.78; 0.84)^**○◊**^	0.89 (0.88; 0.90)	0.83 (0.82; 0.84)	**beta = −0.27**	**beta = −0.06**
Grip strength in kg,^f^*M* (95% CI)	29.6 (28.5; 30.7)	30.7 (30.4; 31.0)	29.1 (28.8; 29.3)	beta = −0.10	beta = 0.05
No. chronic diseases,^e^*M* (95% CI)	1.68 (1.52; 1.84)	1.63 (1.57; 1.68)	1.63 (1.59; 1.67)	beta = −0.04	beta = −0.04
Depressive symptoms,^e^ range 0–60 = worst, *M* (95% CI)	8.87 (7.97; 9.76)^**○◊**^	7.60 (7.31; 7.90)	8.48 (8.26; 8.69)	**beta = −0.17**	**beta = −0.05**
Sense of purpose,^f^ range 0–16 = highest, *M* (95% CI)	11.7 (11.2; 12.1)^**○◊**^	12.3 (12.2; 12.5)	12.3 (12.2; 12.4)	**beta = −0.27**	**beta = −0.26**
Cognitive ability,^e^ range 0–30 = best, *M* (95% CI)	27.1 (26.7; 27.4)	27.1 (27.0; 27.2)	27.0 (26.9; 27.0)	beta = −0.02	beta = 0.03
Lifestyle					
Body mass index,^e^*M* (95% CI)	27.5 (26.8; 28.1)^**◊**^	27.3 (27.1; 27.5)	27.0 (26.9; 27.2)	beta = 0.04	**beta = 0.10**
Waist circumference in cm,^e^*M* (95% CI)	99.3 (97.6; 101.0)^**○◊**^	98.5 (97.9; 99.0)	97.7 (97.3; 98.1)	**beta = 0.07**	**beta = 0.13**
Fruit consumption days/week,^g^*M* (95% CI)	5.9 (5.6; 6.3)^**○◊**^	5.8 (5.7; 5.9)	5.6 (5.5; 5.7)	**beta = 0.07**	**beta = 0.14**
Vegetable consumption days/week,^g^*M* (95% CI)	4.3 (4.0; 4.6)^**○◊**^	4.9 (4.8; 5.0)	4.9 (4.8; 5.0)	**beta = −0.31**	**beta = −0.30**
Alcohol consumption in glasses/week,^e^*M* (95% CI)	4.5 (3.5; 5.5)^**○◊**^	6.5 (6.1; 6.9)	7.2 (6.9; 7.5)	**beta = −0.21**	**beta = −0.29**
Smoking,^e^ % (95% CI)					
-Ever vs never	62 (55; 68)^**○◊**^	72 (70; 74)	76 (74; 78)	**OR = 0.62**	**OR = 0.51**
-Current vs never or past	15 (11; 20)^**◊**^	17 (15; 19)	22 (20; 23)	OR = 0.86	**OR = 0.62**
Walks,^h^ % (95% CI)	75 (69; 81)^**○◊**^	86 (85; 87)	89 (88; 90)	**OR = 0.49**	**OR = 0.38**
Walking in min/day,^h^^,^^i^*M* (95% CI)	20.4 (15.9; 24.9)^**○◊**^	26.9 (25.0; 28.8)	32.3 (30.9; 33.6)	**beta = −0.15**	**beta = −0.27**
Bikes,^h^ % (95% CI)	69 (61; 75)	71 (69; 73)	60 (58; 62)	OR = 0.90	OR = 1.45
Biking in min/day,^h^^,^^i^*M* (95% CI)	18.6 (15.2; 22.0)^**○◊**^	15.7 (14.4; 16.9)	15.2 (14.3; 16.1)	**beta = 0.12**	**beta = 0.14**
Does a sport,^h^ % (95% CI)	44 (36; 51)^**○◊**^	56 (54; 58)	54 (52; 56)	**OR = 0.56**	**OR = 0.57**
Social circle stimulating healthy lifestyle,^d^*M* (95% CI)	42.7 (40.7; 44.8)^**◊**^	42.2 (41.5; 42.8)	41.5 (41.0; 41.9)	beta = 0.04	**beta = 0.11**
Social connectedness					
Partner in household,^e^ % (95% CI)	67 (59; 73)	70 (68; 72)	63 (61; 65)	OR = 0.89	OR = 1.17
Network size,^e^*M* (95% CI)	16.1 (15.0; 17.1)	18.2 (17.7; 18.6)	14.4 (14.1; 14.6)	beta = −0.18	beta = 0.22
Instrumental support received,^e^*M* (95% CI)	14.5 (13.8; 15.1)^**○**^	15.0 (14.8; 15.3)	14.6 (14.4; 14.8)	**beta = −0.08**	beta = −0.02
Emotional support received,^e^*M* (95% CI)	22.6 (21.7; 23.5)^**◊**^	22.4 (22.1; 22.7)	21.1 (20.9; 21.4)	beta = 0.03	**beta = 0.18**
Instrumental support given,^e^*M* (95% CI)	13.7 (13.1; 14.4)^**○◊**^	14.7 (14.4; 14.9)	14.2 (14.0; 14.4)	**beta = −0.15**	**beta = −0.08**
Emotional support given,^e^*M* (95% CI)	22.6 (21.7; 23.5)	23.1 (22.8; 23.4)	21.7 (21.5; 21.9)	beta = −0.12	beta = 0.06
Loneliness,^e^*M* (95% CI)	2.13 (1.83; 2.43)	2.04 (1.93; 2.16)	2.15 (2.07; 2.23)	beta = 0.03	beta = −0.01
Paid work,^e^ % (95% CI)	16 (12; 22)^**○◊**^	10 (9; 12)	10 (9; 11)	**OR = 1.69**	**OR = 1.85**
Volunteering,^e^ % (95% CI)	37 (31; 44)	38 (36; 40)	29 (27; 30)	OR = 0.96	OR = 1.46
Singing,^f^ % (95% CI)	34 (24; 47)^**○◊**^	25 (22; 28)	18 (16; 20)	**OR = 1.59**	**OR = 2.44**
Church attendance > once a month,^j^ % (95% CI)	67 (56; 76)^**○◊**^	42 (39; 45)	24 (22; 26)	**OR = 2.78**	**OR = 6.67**
Strong faith important,^e^ % (95% CI)	33 (27; 40)^**○◊**^	23 (21; 25)	9 (8; 10)	**OR = 1.67**	**OR = 5.26**
Prayer meaningful,^k^ % (95% CI)	88 (80; 93)^**○◊**^	72 (69; 75)	54 (52; 56)	**OR = 2.78**	**OR = 5.88**
Environment					
Walkability total score,^j^*M* (95% CI)	92.5 (92.1; 93.0)^**○◊**^	89.6 (89.3; 89.8)	90.7 (90.5; 90.8)	**beta = 0.38**	**beta = 0.24**
Liveability total score (range 1–7 = best),^j^*M* (95% CI)	5.98 (5.90; 6.05)^**○◊**^	5.73 (5.66; 5.79)	5.05 (4.99; 5.10)	**beta = 0.25**	**beta = 0.93**
Positive neighborhood experience,^e^ % (95% CI)	96 (93; 97)^**○◊**^	91 (90; 92)	84 (83; 85)	**beta = 0.46**	**beta = 0.24**

*Notes*: ApoE e = Apolipoprotein E epsilon; CI = confidence interval; *M* = mean; OR = odds ratio.

^a^From General Estimating Equations with exchangeable correlation matrix and adjusted for year of birth, sex, and educational level; walking, biking, doing a sport, and fruit and vegetable intake are also adjusted for season. The number of observations depends on the number of waves for which a characteristic is available, which range from 1 wave (genetic make-up) to 8 waves (most characteristics).

^b^Indications of significance of differences are based on comparison of the estimate of the Blue Zone with the confidence intervals of the other regions, as follows: ^**○**^ Blue Zone differs from Rest of province; ^**◊**^ Blue Zone differs from Other provinces. Significance is not considered when the Blue Zone has higher values than one region and lower values than the other region.

^c^Effect sizes are marked bold only when a characteristic shows differences between BZ and RP and between BZ and OP in the same direction and when the difference is statistically significant. Effect size for continuous dependent variables: regression coefficient of independent dummy variables comparing RP to BZ and OP to BZ, divided by standard deviation of dependent variable, denoted as beta. Effect size for dichotomous dependent variables: Odds Ratios (ORs) of RP vs BZ and OP vs BZ.

^d^Available for selection of 1 wave (three regions: *N* = 65, 571, and 993, respectively).

^e^Available for 8 waves.

^f^Available for 2 waves.

^g^Available for 3 waves.

^h^Available for 7 waves.

^i^Among participants who walked/biked during the past 2 weeks, additionally adjusted for season and whether the past 2 weeks were normal or not.

^j^Available for 5 waves.

^k^Available for 6 waves.

In the category of physical and mental health, gait speed was significantly lower, depressive symptoms were significantly higher, and sense of purpose was significantly lower in BZ than RP and OP. Effect sizes were small (beta = 0.17–0.27). Other health indicators did not show significant differences.

Mixed results were observed in the category of Lifestyle. In BZ, participants consumed significantly less alcohol and (had) smoked less than RP- and OP-participants, consumed more often fruit than OP-participants, biked for more minutes, and more often had a social circle that stimulated a healthy lifestyle than RP- and OP-participants. For these favorable characteristics, effect sizes were small, ranging from beta = 0.11 (stimulating social circle) to 0.29 (alcohol consumption). The effect size for not smoking was medium (OR = 0.51–0.86). In contrast, body mass index and waist circumference were slightly larger in BZ than in OP-participants and BZ-participants consumed vegetables less often than RP- and OP-participants. Also, they walked less often and spent significantly fewer minutes walking, and a lower proportion practiced any sport than RP- and OP-participants. These unfavorable characteristics had very small to large effect sizes ranging from beta = 0.10 (body mass index) to OR = 0.38 (walks).

Mixed results were also found in the category of social connectedness and participation. The proportion of participants with a partner did not significantly differ between BZ and other regions and the social network size was in-between that of RP and OP. Instrumental support received and given was significantly lower in BZ than elsewhere, but emotional support received was higher in BZ than in OP (small effect size, beta = 0.18). Emotional support given was not significantly different in BZ versus other regions. Regarding participation, a significantly greater proportion of BZ-participants did paid work than RP- and OP-participants (medium effect size, OR = 1.69–1.85), but the proportion of volunteer work in BZ was in between that in RP and OP. A substantially greater proportion in BZ practiced singing, attended church frequently, found a strong faith important, and found prayer meaningful compared to RP and OP. Here, effect sizes were medium to large, with ORs ranging from 1.59 (singing) to 6.67 (church attendance).

Finally, in the category Environment, the total scores of walkability and livability were higher in BZ than in RP and OP. The latter favorable score is echoed in the low percentage of self-reported neighborhood problems in BZ than RP and OP. Effect sizes were small to large, ranging from 0.24 (self-reports) to 0.93 (livability score).

## Discussion and Implications

Since the first identification of limited areas with verified, exceptional longevity by Poulain et al. (2004), who baptized these areas “Blue Zones,” the “Blue Zone” concept is enjoying increasing attention in the scientific literature and frequently emerges in the popular press. The high longevity in BZs has been attributed to various factors that are considered to provide clues to healthy aging. However, the evidence generally does not meet usual scientific quality criteria or addresses only a small selection of factors potentially contributing to longevity. In our study, we compared eleven municipalities across the Netherlands from which the LASA cohorts are sampled and identified one municipality that most closely meets three rigorous criteria to define a BZ. Subsequently, we characterized this municipality using a comprehensive set of variables that have been shown to predict longevity and include all nine BZ “principles” suggested by Buettner and Skemp (2016).

In identifying a BZ, the first criterion was the percentage of exceptionally longevous participants in a municipality. In our study, this is calculated cumulatively over time within the same cohort. As migration most often takes place at young adult ages, Robine and Caselli (2005) recommend to divide the count of centenarians by the count of inhabitants who 40 years earlier were 60 years old. Similarly, in our study, we avoided most migration by ascertaining the percentage of exceptionally longevous participants in a cohort aged 55–85 in 1992. We did this cumulatively up to the end of the study period in 2019 so that annual fluctuations in the number of exceptional longevous participants were avoided. In addition, we ascertained as a criterion the stability of our municipalities using the participants’ place of birth and the moves away from the baseline municipality.

The second criterion for identifying a BZ, longevity, is defined as a continuous measure of survival relative to the survival of the Dutch population of the same birth cohorts and sex, from the LASA baseline onwards. Its added value to the dichotomous measure of reaching a certain age is not only that a dichotomous measure is less informative than a continuous measure, but also that survival may not be positively associated with the number of centenarians (Newman, 2019).

After having identified a BZ, we compared the characteristics of BZ-participants with participants elsewhere in the same province (RP) and with participants of other provinces (OP). BZ-participants were born a few years later than participants in other provinces and had fewer years of schooling, which is generally associated with shorter longevity (Huisman et al., 2013). Both characteristics make the BZ’s relatively high proportion of exceptionally longevous participants even more notable.

Controlling for these sociodemographic characteristics, our findings were mixed. Effect sizes of favorable characteristics were generally small, except for not smoking, doing paid work, singing, religiousness, and livability. For other characteristics, there was no difference or BZ-participants were at a disadvantage. Considering the nine “principles” of BZs, we found unambiguous support only for two: (g) “Faith-based community” and (i) “Right tribe.” Alcohol consumption was even lower than the 1–2 glasses a day implied by principle 6. Curiously, smoking is not included in any of the nine “principles.”

Very few studies exist that compare inhabitants of a well-defined BZ with inhabitants elsewhere in the same region or country on a broad range of characteristics and correcting for potential confounders. Recently, Poulain et al. (2021) compared nonagenarians in two established BZs in Ogliastra (Sardinia, Italy) and Ikaria (Greece) with nonagenarians in other locations in these respective countries. They also found mixed results regarding health status and lifestyle. For example, in both BZs, men smoked more than men in the comparison locations, and alcohol consumption was relatively high in one and relatively low in the other BZ. Poulain and colleagues also studied several genetic markers but did not find clear differences in favor of the BZs. The authors attribute this null finding to the small sample sizes and to the possibility of genetic drift. In our study, the high stability of the BZ population likely contributed to the preservation of its favorable gene pool.

The comparatively strong value attached to religion and prayer in our BZ echoes a study by Fastame et al. (2021), comparing the importance of religion among Sardinian BZ-inhabitants to inhabitants of Sardinia’s capital Cagliari. In the largely secularized Netherlands, it is notable that more than half of the sample considered praying as meaningful. In BZ, this was true for almost nine out of ten participants. Praying is considered as a way to shed stress and has been shown to be associated with longevity (Helm et al., 2000). Singing was another distinctive feature of BZ-participants. As there was a substantial association between singing and church attendance (data not shown), one may assume that much singing was done in the context of church activities.

Clearly, not only individual behavior but also characteristics of the environment were important, showing the largest effect sizes of all characteristics studied. Regarding the physical environment, connectivity was greatest in BZ. Although connectivity is generally considered to indicate the ease of walking between destinations (Hansmann et al., 2024), it may also facilitate biking, as our study shows that in BZ biking was more often practiced than in the comparison regions. BZ was also characterized by having more green space than other regions. Green space has been shown to be a common characteristic of BZs (Herbert et al., 2022). A notably distinctive characteristic of BZ was its livability, as evidenced both by self-reports and a postal code-based composite score. Detailed analysis of the domains of this composite score showed that BZ was particularly characterized by high social cohesion and safety (Supplementary Table 3). To our knowledge, these domains have not been studied in relation to BZs or longevity. However, they tie in with domains studied in the context of concepts such as aging-in-place (Yarker et al., 2023), age-friendly communities (World Health Organization, 2007), and social sustainability (Komp-Leukkunen & Sarasma, 2024). Cross-fertilization between research on these concepts and the BZ concept is recommendable.

### Strengths and Limitations

A strength of our study is the rigorous identification of a BZ and the inclusion of a very broad range of characteristics, with coverage of all nine “principles” of BZs. Regardless, other factors may play an additional role in distinguishing a BZ. For example, optimism and resilience have been found to characterize BZ-inhabitants (Fastame et al., 2021). Also, other operational definitions of the nine “principles” listed by Buettner and Skemp (2016) may show different results. Another strength is a large number of waves available so that our analyses were well powered for most characteristics.

A limitation of our study is that we compared a limited number of municipalities that were selected for the recruitment of the LASA sample. It is not unlikely that a more distinct BZ could be identified elsewhere in the Netherlands. Therefore, we consider the BZ we identified as a relative BZ. Also, BZ is one of the smallest municipalities from which the LASA samples were recruited. Within larger municipalities, there may be districts or neighborhoods that qualify as a BZ.

Furthermore, although we established that the population in BZ was quite stable based on the numbers of participants moving out of the municipality, it is possible that neighboring municipalities did not have sufficient places in care institutions so that older people from elsewhere moved to a care institution in BZ. Hence, they may have added to the number of very old but frail participants. However, this does not seem likely, because care institutions are available in all neighboring municipalities (www.ijsselheem.nl/zorgdiensten), thus reducing the need to seek long-term care elsewhere.

Another limitation is that LASA does not have information on kinship between participants and thus we cannot account for kinship clusters. As the three cohorts were randomly sampled from municipal registries with a low sample density, we consider the number of related participants negligible. Future studies comparing BZs to other regions may specifically address the role of kinship.

Finally, only 1.6% of all participants reported a non-Dutch ethnic background. Future studies should address the role of cultural diversity in the Blue Zone concept.

### Implications

Three main conclusions can be derived from our study. First, contrary to the common attribution, not all aspects of a healthy lifestyle are distinctive for a BZ. Moreover, the health in our BZ is not different from the average health in our study cohort, which represents the sociocultural variety in the Netherlands. This suggests that some characteristics of a BZ may be conducive to a longer life, but this longer life will not necessarily be spent in good health. In this line, Pes et al. (2020) suggested that BZs enable frail individuals to live longer lives despite poor health. Second, a favorable genetic make-up, not smoking, religiousness, and a livable environment appeared to be prominent BZ characteristics. Future research may shed further light on the role of these characteristics in healthy longevity. Third, the truth may be that each municipality or region that is identified as a BZ is characterized by a different set factors, be they genetic, personal, sociocultural, or environmental. This makes it difficult to distill common lessons about aging well from the study of Blue Zones.

## Supplementary Material

gnae132_suppl_Supplementary_Materials

## Data Availability

The committee that evaluates all proposals planning the use of the LASA data, has reviewed and approved this project’s proposal and analysis plan, available from lasa@amsterdamumc.nl. The dataset generated and analyzed are available for replication purposes, provided an agreement is made up. Please see www.lasa-vu.nl. This study was not preregistered.
