# Supplementary material for: Identification of a “Blue Zone” in the Netherlands: A Genetic, Personal, Sociocultural, and Environmental Profile
Source: Gerontologist. 2024 Sep 12;64(11):gnae132. doi: 10.1093/geront/gnae132 (PMC11500713; doi:10.1093/geront/gnae132)
Supplement: gnae132_suppl_Supplementary_Materials [file gnae132_suppl_supplementary_materials.docx]

**Supplementary Material**

**'Identification of a ‘Blue Zone’ in the Netherlands: a genetic, personal, socio-cultural, and environmental profile'**

**Supplement, including a detailed description of the variables used, pertinent references, and three Supplementary tables**

**Measures**

*Socio-demographic covariates*

Age and sex were available from the population registry. Education was assessed in the interview as the highest educational level attained and recoded into number of years (range 5-18).

*Genetics*

Genotyping data were available for 1,629 participants from three cohorts, genotyped using the Illumina Global Screening Array (Tesi et al., 2021). Among genetic variants associated with longevity, two apolipoprotein E (ApoE) alleles, i.e., epsilon-4 (negative association) and epsilon-2 (positive association), have been shown to have the strongest association (Deelen et al., 2019). Participants were classified as epsilon-4 carriers for those with an ApoE epsilon-4 isoform (phenotypes epsilon-2/4, epsilon-3/4, epsilon-4/4) and as epsilon-2 carriers for those with an ApoE epsilon-2 isoform (phenotypes epsilon-2/2, epsilon-2/3, epsilon-2/3). In addition, polygenic risk scores for longevity included 332 Single Nucleotide Polymorphisms (SNPs, p<5x10^-5^) with and 330 SNPs without ApoE variants, derived from a GWAS study on parental longevity (Tesi et al., 2021; Timmers et al., 2019).

*Physical and mental health*

Self-rated health was measured using the question: “How is your health in general”, with scores from 1: very good to 5: poor.

Physical performance was tested as walking speed and grip strength, two key components of physical performance with unique predictive value for mortality (Nofuji et al., 2016). For the walk test, participants were instructed to walk 3 meters, turn around, and walk 3 meters back as quickly as possible. The time used was recorded to the nearest second, and converted to gait speed in meters/seconds. Handgrip strength was measured to the nearest 1 kg with a handheld dynamometer (Takei TKK 5001, Takei Scientific Instruments Co. Ltd., Tokyo, Japan), which was adjusted for the hand size of the participant. Measurements were done in duplicate for both hands; the maximum of each hand was used to calculate the average grip strength.

The number of chronic diseases was assessed by self-reports. Seven diseases were explicitly asked: chronic non-specific lung disease, cardiac disease, peripheral arterial disease, diabetes mellitus, stroke, arthritis, and cancer. In addition, participants were asked to report a maximum of two chronic diseases that were not explicitly asked. The number of chronic diseases ranged from 0-9.

Depressive symptoms were ascertained using the Dutch translation of the 20-item Center for Epidemiologic Studies Depression scale (CES-D, Radloff, 1977; Beekman et al., 1997). Participants were asked to indicate how often during the past week they had experienced each symptom with response categories 0: (almost) never to 3: (almost) always. The score range was 0-60.

Sense of purpose was assessed using the 4-item Ambition subscale of the Valuation of Life scale (Lawton et al., 2001). This subscale included: Original item 11, My personal beliefs allow me to maintain a hopeful attitude; Original item 14, I have a strong will to live right now; Original item 16, I intend to make the most of my life; and Original item 18, Life has meaning for me. The subscale was shown to be reliable with Cronbach’s alpha 0.80 (Knipscheer et al., 2008). Items were rated on a 5-point scale. The score ranged from 0: lowest to 16: highest sense of purpose.

Cognitive ability was measured using the Mini-Mental State Examination. Scores ranged from 0-30, with higher scores indicating better cognitive functioning (Folstein et al., 1975).

*Lifestyle*

Body Mass Index (BMI) was calculated as measured weight in kg over squared measured height in m. Waist circumference (in cm) was calculated as the mean of two measurements in standing position, midway between the lower rib and the iliac crest after normal expiration.

Fruit and vegetable intake was assessed using a questionnaire in which participants were asked on how many days of the week they consumed fruits and vegetables (Dijkstra et al., 2014; Visser et al., 2020). Intakes during spring/summer and fall/winter were averaged.

Past and current smoking status was assessed by asking the participants whether they had ever smoked and whether they currently smoked or not. Alcohol consumption was assessed using two questions: on how many days per week alcoholic beverages were consumed and how many glasses each time. The responses were multiplied to obtain the number of glasses per week (Reinders et al., 2018).

Habitual physical activity was measured using the LASA Physical Activity Questionnaire (Stel et al., 2004), which covers frequency and duration of various activities during the previous two weeks. Selected activities include walking outside, bicycling, and doing a maximum of two sports. Among participants who walked and biked, the average daily physical activity in minutes per day were calculated by multiplying the frequency and duration of each activity, divided by 14 days. Doing a sport was defined as a dichotomous variable. An additional question was whether the past two weeks were normal or not.

Finally, to operationally define the ‘Right tribe’ principle, questions were asked about the extent to which: 1) important social contacts have a healthy lifestyle, 2) important social contacts stimulate the participant’s healthy lifestyle, 3) the participant deems a healthy lifestyle important. Response categories ranged from 1: most negative to 5: most positive. These three questions were asked for physical activity, weight, nutrition, and lifestyle in general. All twelve questions were summed to a scale with range 12 to 60. This scale proved to be reliable with Cronbach’s alpha 0.83.

*Social connectedness and participation*

Partner status was categorized as 0: no partner or partner outside the household and 1: living with partner. Personal network size was based on the total number of persons with whom the participant had frequent and meaningful contact (range 0-80). Social support was assessed as the instrumental and emotional support received and given from the nine most frequently contacted persons, coded as 1: never to 4: often, and summed to a maximum of 36 (van Tilburg, 1998). Employment participation was defined as having a paid job of one hour or more weekly. Volunteering work was defined using two questions, one indicating involvement in clubs or organizations, and the next, among participants who reported involvement, distinguishing board membership and volunteering. They were coded as 0: no member or not on board/no volunteer and 1: board member/volunteer (Broese van Groenou & Deeg 2010). Singing as a hobby was also coded as 0: no and 1: yes (Mansens et al., 2018). Church attendance was coded as 0: once a month or less, 1: more often than once a month. The salience of religion was assessed by the importance a participant attached to a strong faith in relation to eight other domains of life, such as good health and good housing. When ‘a strong faith’ was selected among the three most important domains, it was considered to be salient (Braam et al., 1997). Finally, participants were asked if they deemed praying meaningful, a dichotomous variable (Braam et al., 2007).

*Environment*

Data on walkability and livability of the home environment were derived from the Geoscience and Health Cohort Consortium database, covering the years 1998 through 2016 (Lakerveld et al., 2020; Timmermans et al., 2018). Walkability was defined in a circle (buffer zone) around each address of 500 meter, which is a commonly used buffer size (Barnett et al., 2017). Walkability includes six items that have been shown to be associated with walking activity: population density (number of inhabitants per hectare in buffer zone), density of retail and services (percentage in buffer zone), land use mix (range 0-1, with 1 representing perfect mix of all relevant land uses in buffer zone), connectivity (number of street intersections accessible for pedestrians in buffer zone), side walk density (percentage in buffer zone), and green space (percentage in buffer zone) (Timmermans et al., 2021). The walkability total score was calculated by summing the z-scores of these six components, and then min-max scaling the result such that it ranged from 0 to 100, with 100 representing the highest walkability. The livability index consists of six dimension scores, which are derived from 49 indicators: population composition (6 items), social cohesion (11 items), public space (8 items), safety (5 items), level of resources (3 items), and housing stock (13 items). The livability total score ranges from 1: extremely negative to 7: extremely positive (Leidelmeijer et al., 2011).

In addition, in the LASA interview participants were asked whether they generally enjoyed living in their neighborhood and whether they felt safe in the evening in their neighborhood, with response options ‘no’ and ‘yes’. These variables were combined into one variable, coded as 0: not pleasant and/or not safe and 1: pleasant and safe.

**References**

Barnett, D. W., Barnett, A., Nathan, A., Van Cauwenberg, J., & Cerin, E., on behalf of the Council on Environment and Physical Activity (CEPA) – Older Adults working group (2017). Built environmental correlates of older adults’ total physical activity and walking: a systematic review and meta-analysis. *International Journal of Behavioral Nutrition and Physical Activity*, **14**, 103. https://doi.org/10.1186/s12966-017-0558-z

Beekman, A. T. F., Deeg, D. J. H., van Limbeek, J., Braam, A. W., de Vries, M. Z., & van Tilburg, W. (1997). Criterion validity of the Center for Epidemiologic Studies Depression scale (CES-D): Results from a community based sample of older subjects in the Netherlands. *Psychological Medicine*, **27**, 231-235. https://doi.org/10.1017/s0033291796003510

Braam, A. W., Beekman, A. T. F., Deeg, D. J. H., Smit, J. H., van Tilburg, W. (1997). Religiosity as a protective or prognostic factor of depression in older adults; results from a community survey in the Netherlands. *Acta Psychiatrica Scandinavica*, **96**, 199-205. https://doi.org/10.1111/j.1600-0447.1997.tb10152.x

Braam, A. W., Deeg, D. J. H., Poppelaars, J. L., Beekman, A. T. F., & van Tilburg, W. (2007). Prayer and depressive symptoms in a period of secularization: patterns among older adults in the Netherlands. *American Journal of Geriatric Psychiatry*, **15**, 273-281. https://doi.org/10.1097/JGP.0b013e31802d0ae8

Broese van Groenou, M. I., & Deeg, D. J. H. (2010). Formal and informal social participation of the 'young-old' in The Netherlands in 1992 and 2002. *Ageing & Society*, **30**, 445-465. https://doi.org/10.1017/S0144686X09990638

Deelen, J., Evans, D. S., Arking, D. E., Tesi, N., Nygaard, M., Liu, X., Wojczynski, M. K., Biggs, M. L., van der Spek, A., Atzmon, G., Ware, E. B., Sarnowski, C., Smith, A. V., Seppälä, I., Cordell, H. J., Dose, J., Amin, N., Arnold, A. M., Ayers, K. L., … Murabito, J. M. (2019). A meta-analysis of genome-wide association studies identifies multiple longevity genes. *Nature Communications*, **10**, 3669. https://doi.org/10.1038/s41467-019-11558-2

Dijkstra, S. C., Neter, J. E., Brouwer, I. A., Huisman, M., & Visser, M. (2014). Adherence to dietary guidelines for fruit, vegetables and fish among older Dutch adults; the role of education, income and job prestige. *Journal of Nutrition Health and Aging*, **18**(2), 115-121. https://doi.org/10.1007/s12603-013-0402-3

Folstein, M. F., Folstein, S. E., & McHugh, P. R. (1975). Mini-mental state: a practical method for the clinician. *Journal of Psychiatric Research* 1975; **12**(3), 189-198. https://doi.org/10.1016/0022-3956(75)90026-6

Knipscheer, K., van Schoor, N. M., Penninx, B., & Smit, J. H. (2008). Adaptation and validation of the Dutch translation of the “Valuation of Life” scale. *Tijdschrift voor Gerontologie en Geriatrie*, **39**(4), 133-145. https://doi.org/10.1007/BF03078142 In Dutch

Lakerveld, J., Wagtendonk, A., Vaartjes, I., Karssenberg, D., & GECCO consortium (2020). Deep phenotyping meets big data: the Geoscience and hEalth Cohort COnsortium (GECCO) data to enable exposome studies in the Netherlands. *International Journal of Health Geography*, **19**(1), 49. https://doi.org/10.1186/s12942-020-00235-z

Lawton, M. P., Moss, M., Hoffman, C., Kleban, M. H., Ruckdeschel, K., & Winter, L. (2001). Valuation of Life: a concept and a scale. *Journal of Aging and Health*, **13**(1), 3-31. https://doi.org/10.1177/089826430101300101

Leidelmeijer, K., Marlet, G., van der Reijden, H., van Woerkens, C., & Schulenberg, R. (2011). *Leefbaarometer [Liveability measurement] – update 2010*. RIGO Research en Advies. In Dutch.

Mansens D., Deeg D. J. H., & Comijs H. C. (2018). The association between singing and/or playing a musical instrument and cognitive functions in older adults. *Aging & Mental Health*, **22**(8), 964-971. https://doi.org/10.1080/13607863.2017.1328481

Nofuji, Y., Shinkai, S., Taniguchi, Y., Amano, H., Nishi, M., Murayama, H., Fujiwara, Y., & Suzuki, T. (2016). Associations of walking speed, grip strength, and standing balance with total and cause-specific mortality in a general population of Japanese elders. *Journal of the American Medical Directors Association*, **17**, 184.e1-184.e7. https://doi.org/10.1016/j.jamda.2015.11.003

Radloff, L. S. (1977). The CES-D scale: a self-report depression scale for research in the general population. *Applied Psychology Measurement*, **1**(3), 385-401. https://doi.org/10.1177/014662167700100306

Reinders, I., van Schoor, N. M., Deeg, D. J. H., Huisman, M., & Visser, M. (2018). Trends in lifestyle among three cohorts of adults aged 55-64 years in 1992/1993, 2002/2003 and 2012/2013. *European Journal of Public Health*, **28**(3), 564-570. https://doi.org/10.1093/eurpub/ckx173

Stel, V. S., Smit, J. H., Pluijm, S. M., Visser, M., Deeg, D. J., & Lips, P. (2004). Comparison of the LASA Physical Activity Questionnaire with a 7-day diary and pedometer. *Journal of Clinical Epidemiology*, **57**(3), 252-258. https://doi.org/10.1016/j.jclinepi.2003.07.008

Tesi, N., van der Lee, S. J., Hulsman, M., Jansen, I. E., Stringa, N., van Schoor, N. M., Scheltens, P., van der Flier, W. M., Huisman, M., Reinders, M. J. T., & Holstege, H. (2021). Polygenic risk score of longevity predicts longer survival across an age continuum. *Journals of Gerontology, Series A: Biological Sciences Medical Sciences*, **76**(5), 750-759. https://doi.org/10.1093/gerona/glaa289

Timmermans, E. J., Lakerveld, J., Beulens, J. W. J., Boomsma, D. I., Kramer, S. E., Oosterman, M., Willemsen, G., Stam, M., Nijpels, G., Schuengel, C., Smit, J. H., Brunekreef, B., Dekkers, J. E. C., Deeg, D. J. H., Penninx, B. W. J. H., & Huisman, M. (2018). Cohort profile: the Geoscience and Health Cohort Consortium (GECCO) in the Netherlands. *BMJ Open*, **8**(6), e021597. https://doi.org/10.1136/bmjopen-2018-021597

Timmermans, E. J., Visser, M., Wagtendonk, A. J., Noordzij, J. M., & Lakerveld, J. (2021). Associations of changes in neighbourhood walkability with changes in walking activity in older adults: a fixed effects analysis. *BMC Public Health*, **21**, 1323. https://doi.org/10.1186/s12889-021-11368-6

Timmers, P. R., Mounier, N., Lall, K., Fischer, K., Ning, Z., Feng, X., Bretherick, A. D., Clark, D. W., eQTLGen Consortium, Shen, X., Esko, T., Kutalik, Z., Wilson, J. F., Joshi, P. K. (2019). Genomics of 1 million parent lifespans implicates novel pathways and common diseases and distinguishes survival chances. *eLife*, **8**, e39856. doi:10.7554/eLife.39856

van Tilburg, T. G. (1998) Losing and gaining in old age: Changes in personal network size and social support in a four-year longitudinal study. *Journal of Gerontology: Social Sciences*, **53B**(6), S313-S323. https://doi.org/10.1093/geronb/53b.6.s313

Visser, M., Elstgeest, L. E. M., Winkens, L. H. H., Brouwer, I. A., & Nicolau, M. (2020). Relative validity of the HELIUS Food Frequency Questionnaire for measuring dietary intake in older adult participants of the Longitudinal Aging Study Amsterdam. *Nutrients*, **12**(7), 1998. https://doi.org/10.3390/nu12071998

**Attrition across waves and municipalities**

Table S1 presents wave-to-wave attrition by municipality and statistical significance of change over the study period, calculated as marginal estimates from General Estimating Equations without covariates. Table S2 presents the effects of sex, year of birth, and years of education on attrition.

**Table S1.** Number of participants in sample at recruitment and % wave-to-wave attrition due to all causes and non-mortality attrition for each municipality

| **Municipality** | **N at recruitment^a^** | **All-cause attrition** | | **Non-mortality attrition** | |
| --- | --- | --- | --- | --- | --- |
|  |  | % | p-value for change | % | p-value for change |
| N1 | 168 | 16.8 | 0.183 | 6.5 | 0.796 |
| N2 | 162 | 16.4 | 0.900 | 5.7 | 0.180 |
| N3 | 423 | 17.3 | 0.870 | 6.3 | <0.001^b^ |
| N4 | 286 | 17.2 | 0.654 | 5.6 | 0.005^b^ |
| N5 | 601 | 17.2 | 0.132 | 5.4 | 0.029^b^ |
| W1 | 1359 | 19.5 | <0.001^c^ | 6.2 | 0.002^b^ |
| S1 | 248 | 16.6 | 0.080 | 5.1 | 0.416 |
| S2 | 612 | 19.4 | 0.002^c^ | 6.6 | <0.001^b^ |
| W2 | 437 | 16.4 | <0001^c^ | 4.4 | 0.205 |
| S3 | 344 | 19.7 | <0.001^c^ | 6.2 | 0.119 |
| W3 | 454 | 16.7 | <0.001^c^ | 5.5 | 0.284 |
| Total | 5094 | 18.0 | 0.014^c^ | 5.8 | 0.170 |

^a^ Recruited at baseline (1992), in 2002, or in 2012. The total number is 38 lower than the sample size, because 38 participants moved outside the 11 municipalities between sampling and baseline interview. These 38 participants were included in the comparison of BZ and other regions.

^b^ Increased over study period

^c^ Decreased over study period

The time lag between waves was three or four years by design. The first four waves of each cohort, covering nine years, have a time lag of three years. The new cohorts that are recruited in 2002/03 and 2012/13 are merged with the earlier cohorts after three years, so that the earlier cohorts have a time lag of four years between the fourth and fifth and between the eighth and ninth wave. Given this design, the time lag varies somewhat between participants, with a standard deviation of 86 days for both the 3-year and 4-year time lag.

Overall (last line ‘Total’ in Table S1), all-cause attrition decreases across the study period. As mortality is the main driver of attrition, this decrease parallels the substantial decline in mortality in the general population mortality. Overall, non-mortality attrition does not statistically significantly decrease or increase, although in five municipalities it statistically significantly increases. Because these five municipalities are spread across different regions (N, W, and S), the differences in change in non-mortality attrition are unlikely to affect the comparison across regions.

**Table S2**. Factors affecting wave-to-wave attrition

|  | **All-cause attrition** | | **Non-mortality attrition** | |
| --- | --- | --- | --- | --- |
|  | Odds Ratio | 95% Confidence interval | Odds Ratio | 95% Confidence interval |
| Sex (male vs female) | 1.50 | 1.38; 1.62 | 1.01 | 0.89; 1.15 |
| Year of birth | 0.92 | 0.92; 0.93 | 1.03 | 1.02; 1.03 |
| Years of education | 0.95 | 0.94; 0.96 | 0.93 | 0.91; 0.95 |
| Municipality (ref. = W3) |  |  |  |  |
| - N1 | 1.02 | 0.78; 1.33 | 1.05 | 0.71; 1.57 |
| - N2 | 1.00 | 0.78; 1.27 | 0.95 | 0.63; 1.43 |
| - N3 | 1.12 | 0.92; 1.36 | 1.14 | 0.84; 1.53 |
| - N4 | 1.02 | 0.92; 1.36 | 0.92 | 0.66; 1.30 |
| - N5 | 1.08 | 0.90; 1.29 | 1.01 | 0.76; 1.34 |
| - W1 | 1.21 | 1.04; 1.42 | 1.23 | 0.95; 1.58 |
| - S1 | 0.96 | 0.77; 1.21 | 0.87 | 0.60; 1.27 |
| - S2 | 1.23 | 1.03; 1.47 | 1.21 | 0.92; 1.60 |
| - W2 | 0.94 | 0.78; 1.13 | 0.80 | 0.58; 1.11 |
| - S3 | 1.25 | 1.01; 1.55 | 1.16 | 0.84; 1.62 |

^a^

All-cause attrition ranges from 16.4 to 19.7%. Differences among municipalities are statistically significant (p = 0.014). This remains the case when sex, year of birth, and years of education are included as covariates (p = 0.006). Again, as mortality is the main driver of all-cause attrition, the differences among municipalities roughly correspond to the differences in relative survival time (Table 2 of main document). As observed in Table S2, all three covariates are statistically significantly associated with all-cause attrition.

Non-mortality attrition ranges from 4.4 to 6.6% across municipalities. Differences among municipalities are not statistically significant (p = 0.170). This remains the case when sex, year of birth, and years of education are included as covariates (p = 0.064). As observed in Table S2, year of birth and years of education, but not sex, are statistically significantly associated with all-cause attrition.

**Table S3**. Detailed environmental characteristics^a,b^ of Blue Zone compared to the rest of its province and other provinces

|  | **Blue Zone (BZ)** (n=330) | **Rest of province (RP)** (n=3,118) | **Other provinces (OP)** (n=6,793) | **Effect size^c^**  **BZ - RP** | **Effect size^c^**  **BZ - OP** |
| --- | --- | --- | --- | --- | --- |
| ***Walkability domains*** | | | |  |  |
| Population density (inhabitants per hectare in buffer zone), M (95%CI) | 34.2 (32.2; 36.2) | 29.1 (27.4; 30.9) | 52.5 (50.7; 54.3) | beta=0.15 | beta=-0.49 |
| Availability of retail & services (% of buffer zone), M (95%CI) | 1.7 (1.4; 2.0)**^○◊^** | 4.0 (3.7; 4.3) | 4.3 (4.1; 4.5) | **beta=-0.48** | **beta=-0.56** |
| Land use mix (range 0-1), M (95%CI) | 0.35 (0.34; 0.35)**^○◊^** | 0.38(0.37; 0.39) | 0.36 (0.36; 0.37) | **beta=-0.28** | **beta=-0.12** |
| Connectivity (number of intersections accessible for pedestrians in buffer zone), M (95%CI) | 1.5 (1.5; 1.6)**^○◊^** | 1.0 (1.0; 1.1) | 1.1 (1.1; 1.2) | **beta=1.17** | **beta=0.91** |
| Sidewalk density (% of buffer zone), M (95%CI) | 5.0 (4.6; 5.4) | 4.4 (4.0; 4.7) | 7.7 (7.4; 7.9) | beta=0.16 | beta=-0.64 |
| Green space (% of buffer zone), M (95%CI) | 8.3 (6.8; 9.8)**^◊^** | 8.0 (7.6; 8.4) | 7.2 (7.0; 7.5) | **beta=0.07** | **beta=0.21** |
| ***Livability domains*** | | | |  |  |
| Population (range -50 to +50), M (95%CI) | 21.9 (20.2; 23.7)**^○◊^** | 12.5 (11.0; 14.0) | -0.2 (-1.3; 1.0) | **beta=0.42** | **beta=0.98** |
| Social cohesion (range -50 to +50), M (95%CI) | 11.4 (10.0; 12.9)**^○◊^** | 3.4 (2.6; 4.2) | 0.6 (0.1; 1.2) | **beta=0.57** | **beta=0.77** |
| Public space (range -50 to +50), M (95%CI) | 7.7 (2.0; 13.5) | 3.9 (2.0; 5.8) | 17.1 (16.0; 18.1) | beta=0.17 | beta=-0.41 |
| Safety (range -50 to +50), M (95%CI) | 33.1 (30.5; 35.7)**^○◊^** | 19.3 (17.2; 21.5) | -1.2 (-2.8; 0.3) | **beta=0.48** | **beta=1.20** |
| Resources (range -50 to +50), M (95%CI) | -33.3 (-35.2; -31.3)**^○◊^** | -11.9 (-13.4; -10.4) | 3.8 (2.8; 4.8) | **beta=-0.79** | **beta=-1.41** |
| Housing (range -50 to +50), M (95%CI) | 20.3 (15.9; 24.6)**^○◊^** | 7.0 (4.7; 9.4) | -9.3 (-10.8; -7.8) | **beta=0.48** | **beta=1.07** |

Abbreviations: M=mean; CI=confidence interval; OR=Odds Ratio

**^a^** Derived from the Geoscience and Health Cohort Consortium database, covering the years 1998 through 2016 (Lakerveld et al., 2020; Timmermans et al., 2018)

^b^ From General Estimating Equations with exchangeable correlation matrix and adjusted for year of birth, sex, and educational level. Indications of significance of differences are based on comparison of the estimate of the Blue Zone with the confidence intervals of the other regions, as follows: **^○^** Blue Zone differs from Rest of province; **^◊^** Blue Zone differs from Other provinces. Significance is not considered when the Blue Zone has higher values than one region and lower values than the other region.

^c^ Effect sizes are marked bold only when a characteristic shows differences between BZ and RP and between BZ and OP in the same direction and when at least one difference is statistically significant. Effect size is calculated as the regression coefficient of independent dummy variables comparing RP to BZ and OP to BZ, divided by standard deviation of the dependent variable, denoted as beta.
